# Supplementary material for: CLEP-GAN: an innovative approach to subject-independent ECG reconstruction from PPG signals
Source: BMC Bioinformatics. 2025 Nov 25;26:306. doi: 10.1186/s12859-025-06276-0 (PMC12751419; doi:10.1186/s12859-025-06276-0)
Supplement: Supplementary file 1 — Supplementary Material 1 [file 12859_2025_6276_MOESM1_ESM.pdf]

Appendix A Adversarial Learning

Within the realm of generative modelling, GANs have emerged as a pioneering framework, enabling the generation of high-quality data that closely resembles real-world samples. GANs consist of two key components: a generator and a discriminator. The generator crafts synthetic data samples, while the discriminator evaluates whether a given sample is real or generated. Through an adversarial process, the generator refines its outputs to become progressively more convincing, while the discriminator enhances its ability to differentiate between real and synthetic data. This dynamic interplay interplay drives continuous improvement, ultimately resulting in the generation of data that aligns closely with real observations. In our pursuit to enhance the ECG reconstruction process, we harness the potency of GAN-based adversarial learning. By integrating GAN architecture into our generator network, we empower it to yield reconstructed ECG signals with high accuracy.

To maintain fidelity to both time and frequency characteristics of cardiac dynamics, we adopt a dual discriminator strategy [15, 60]. To utilize a frequency-domain-based discriminator, we first need to transform the signals from the time domain to the frequency domain. This approach involves employing the Short-Time Fourier Transformation (STFT) on the ECG and PPG time series data. Denoting the time series as $x[n]$, the STFT on $x[n]$, denoted as $STFT\{x[n]\}(m,\omega) \equiv X(m,\omega) = \sum_{n=-\infty}^{\infty} x[n]w[n-m]e^{-i\omega n}$, captures the data's spectral content, where m signifies the step size and $w[n]$ represents the Hann window function. The spectrogram is ultimately derived from $STFT_{spect}(x[n]) = \log(|X(m,\omega)| + \delta)$, where δ is a small number added to avoid potential infinite conditions.

Appendix B Attention U-Net

The Attention U-Net incorporates an attention gate (AG) into the standard U-Net architecture. Fig. B2 illustrates the architecture of the Attention U-Net, emphasizing its capacity to concentrate on vital features within the skip connections. Within this structure, features denoted by x_i^l are sourced from the skip connection at layer l. The gating vector, symbolized by g, designates the focal region. These features and the gating vector are mapped to an intermediate-dimensional space $R^{F_{\text{int}}}$, with F_{int} specifying the dimensions of this space. The objective is to derive scalar attention values, α_i^l, for each temporal unit $x_i^l \in R^{F_l}$ based on the gating vector $g_i \in R^{F_g}$.

To achieve this, linear transformations are applied to x_i^l and g_i using weights and biases, denoted as $\theta_x = W_x x_i^l + b_x$ and $\theta_g = W_g g_i + b_g$, respectively. Here, $W_x \in R^{F_l \times F_{\text{int}}}$, $W_g \in R^{F_g \times F_{\text{int}}}$, and b_x, b_g are the bias terms. Following these transformations, a non-linear ReLU activation (denoted as σ_1) is applied to yield a summed feature activation $f = \sigma_1(\theta_x + \theta_g)$.

Subsequently, a linear mapping of f onto the $R^{F_{\text{int}}}$ dimensional space occurs through channel-wise 1×1 convolutions. The result is passed through a sigmoid activation function (σ_2) to obtain attention weights. The attention map for x_i^l is derived as $\alpha_i^l = \sigma_2(\psi * f)$, where $\psi \in R^{F_{\text{int}}}$ and $*$ denotes convolution. Finally, we perform element-wise multiplication between x_i^l and α_i^l to yield the ultimate output from the attention layer.

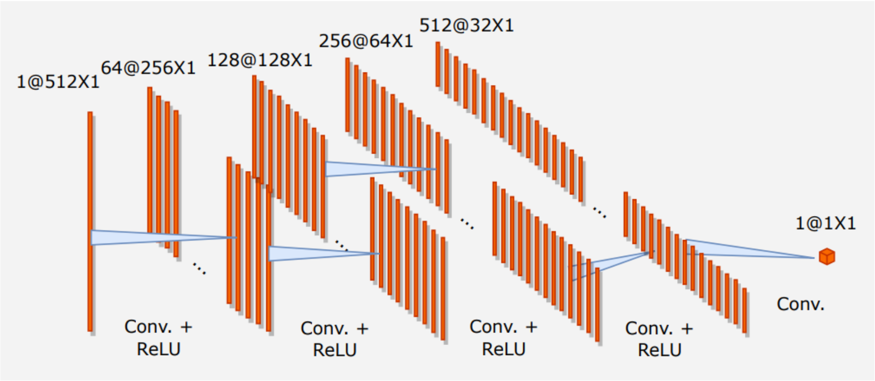

(a) Time domain-based discriminator.

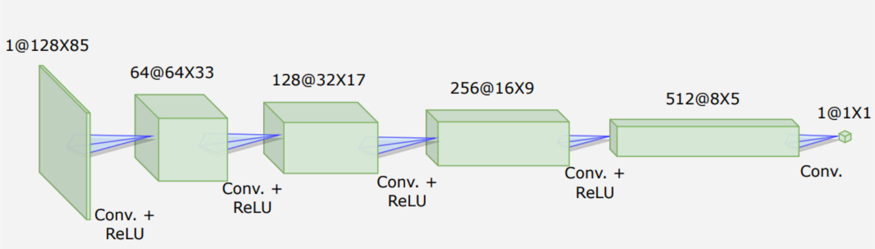

(b) Frequency domain-based discriminator.

Fig. A1: Architectures of dual discriminators. The format "C@HxW" describes the dimensions of a feature layer. "C" stands for the number of channels, "H" indicates the height of the feature map, and "W" specifies its width. For instance, "1@512x1" refers to a layer with a single channel, a height of 512, and a width of 1.

.

Appendix C Discrete Latent variables

The authors in [40] introduced the concept of VQ-VAE, which employs discrete latent variables and utilizes a novel training methodology inspired by vector quantization (VQ). In this structure, both posterior and prior distributions are categorical, and the samples extracted from these distributions are used to index an embedding table. These indexed embeddings subsequently serve as inputs for the decoder network.

A latent embedding space is delineated as $e \in \mathbb{R}^{K \times D}$, where K signifies the size of the discrete latent space, represented as a K-way categorical variable, and D denotes the dimensionality of each latent embedding vector e_i. It should be noted that there are K embedding vectors denoted by $e_i \in \mathbb{R}^D$, where $i = 1, 2, \ldots, K$. The model accepts an input x that traverses through an encoder, yielding an output $z_e(x)$. Following this, the discrete latent variables z are computed using a nearest-neighbor lookup mechanism that employs the shared embedding space e, as illustrated in the posterior categorical distribution $q(z|x)$ probabilities:

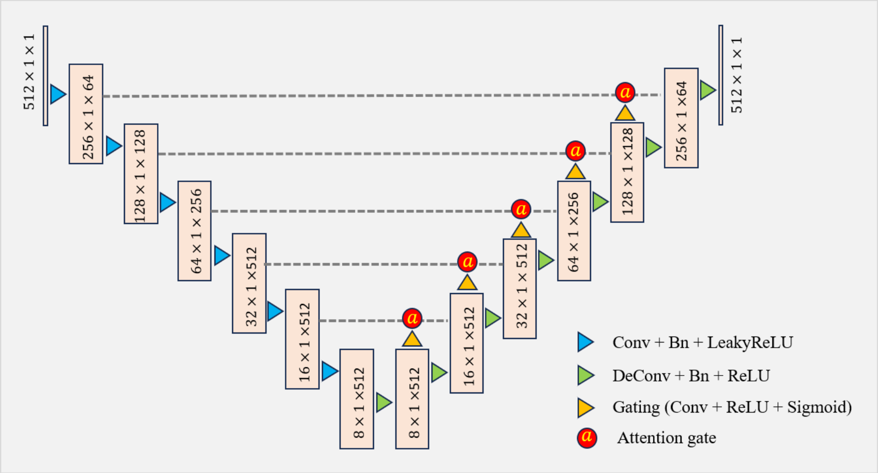

Fig. B2: The architecture of Attention U-Net generator.

$$q(z=k|x) = \begin{cases} 1 & \text{if } k = \text{argmin}_i \|z_e(x) - e_i\|_2, \\ 0 & \text{otherwise.} \end{cases} \tag{C1}$$

In this scenario, a singular random variable z is utilized to represent the discrete latent variables for simplicity. The decoder receives the respective embedding vector e_k as denoted by $z_q(x) = e_k$, where $k = \text{argmin}_j \|z_e(x) - e_j\|_2$. This forward computation pathway can be perceived as a standard autoencoder, characterized by a unique non-linearity that aligns the latent variables to one of the K embedding vectors. Viewing this model through the lens of a VAE, it is observed that $q(z = k|x)$ operates deterministically. Furthermore, by establishing a uniform prior over z, the KL divergence remains consistent, equating to $\log K$.

Appendix D Inspecting the Deviation of RR Intervals

As previously discussed, when using RMSE as the evaluation metric, the performance of the original CardioGAN lags behind that of other methods. Additionally, Fig. 7 illustrates that it faces more challenges in reconstructing small waveforms compared to other methods. However, as indicated in Table 5, CardioGAN showcases impressive accuracy in HRV, surpassing all other methods.

To visualize the RR intervals, we have plotted the ground truth RR interval distribution of signal 0332 in CapnoBase, as shown in Fig. D5. We have also included several cycles of reconstructed ECGs labeled with corresponding RR intervals from five different methods, as displayed in Fig. D6.

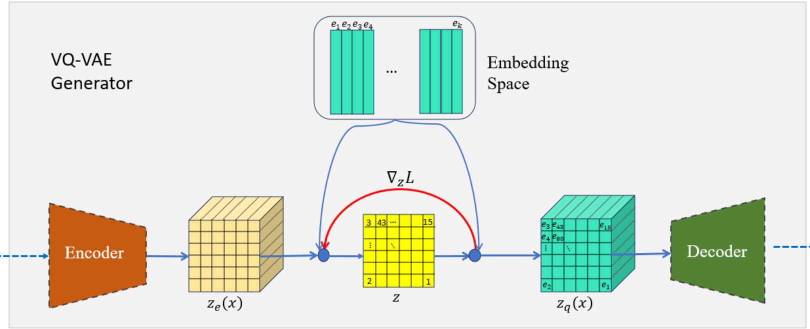

Fig. C3: The architecture of VQ-VAE. The encoder output, denoted as $z_e(x)$, is mapped to the nearest points, e_i, within the embedding space. During forward computation, the nearest embedding, $z_q(x)$, is passed to the decoder, and during the backward pass the gradient, $\nabla_z L$, is transmitted unaltered back to the encoder.

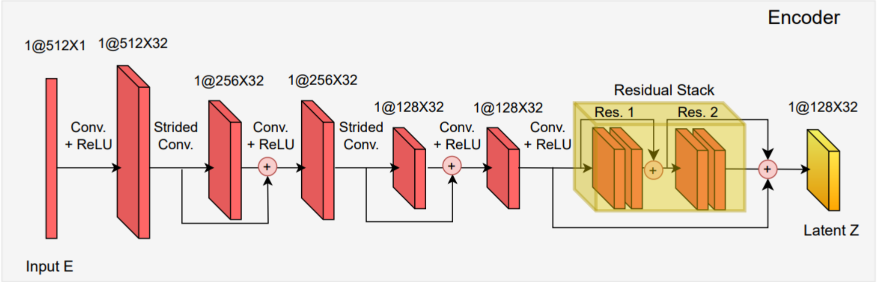

(a) Encoder of VQ-VAE.

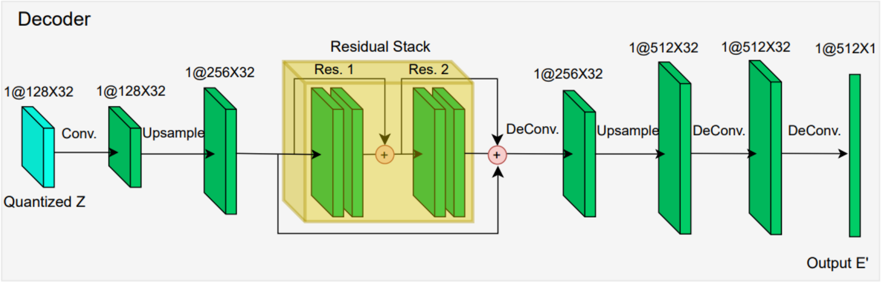

(b) Decoder of VQ-VAE.

Fig. C4: A depiction of the VQ-VAE's encoder and decoder architectures. Both the encoder and decoder utilize residual stacks, each comprising two residual blocks.

.

From Fig. D5, it is evident that the RR interval distribution of signal 0332 exhibits a bimodal shape, resembling two connected Gaussian distributions. However, there

are several RR intervals significantly distant from the left Gaussian-like distribution, resulting in a large standard deviation of the RR intervals.

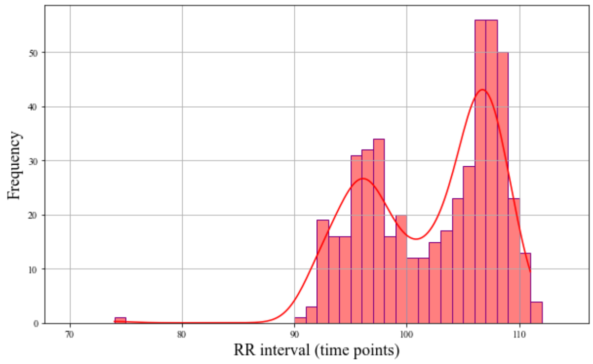

Fig. D5: RR distribution of signal 0332 in the CapnoBase dataset. RR intervals are measured in data points.

Analyzing Fig. D6, we observe that the reconstructed ECGs using CardioGAN consistently exhibit a leftward shift, leading to a higher RMSE. An interesting observation is that, although each RR interval does not exactly match the corresponding ground truth values, the model attempts to balance the RR interval distribution. For instance, in the case of CardioGAN, when the first RR interval of the reconstructed ECG is 824 ms, whereas its ground truth has a smaller interval of 808 ms, the second RR interval of the reconstructed ECG becomes 752 ms, which is 16 ms shorter than its ground truth, in an attempt to achieve balance. This balancing effect is noticeable in the reconstructed ECGs of other methods as well.

The sex and age distribution of the ECG-PPG pairs used in our experiments is depicted in Fig. D7.

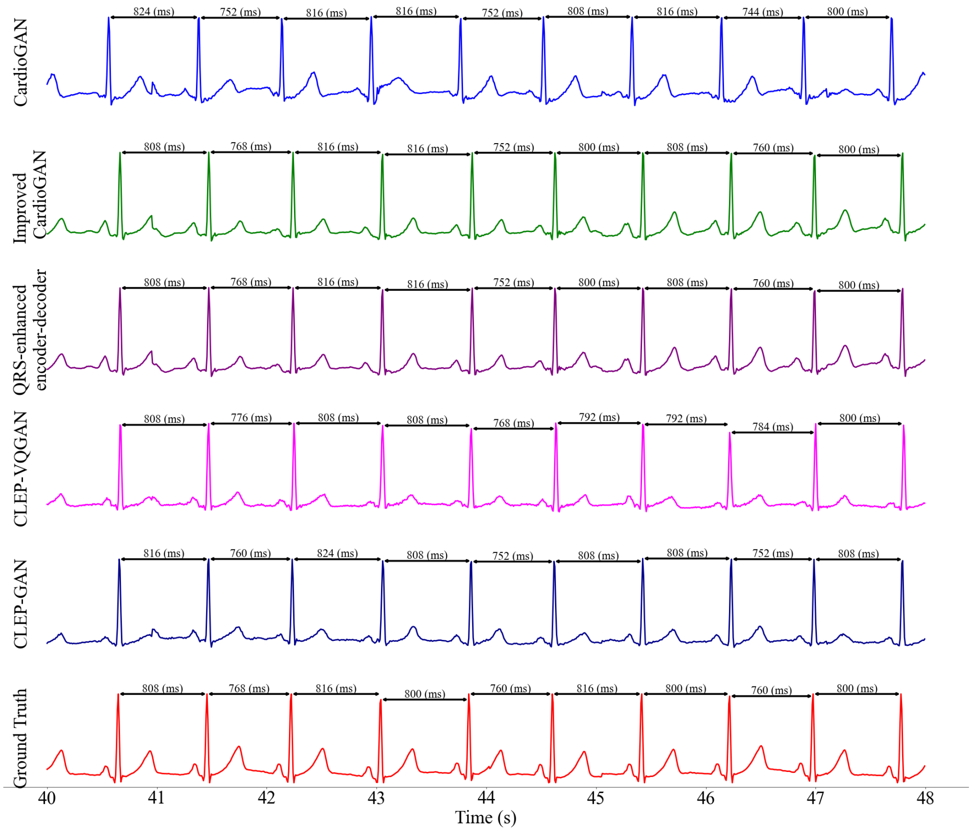

Fig. D6: Cycles of the reconstructed signal 0332 from the CapnoBase dataset labeled with RR intervals. ECGs were reconstructed using five methods: three proposed (improved CardioGAN, CLEP-VQGAN, and CLEP-GAN) and two advanced (CardioGAN [15] and QRS complex-enhanced encoder-decoder [8]).

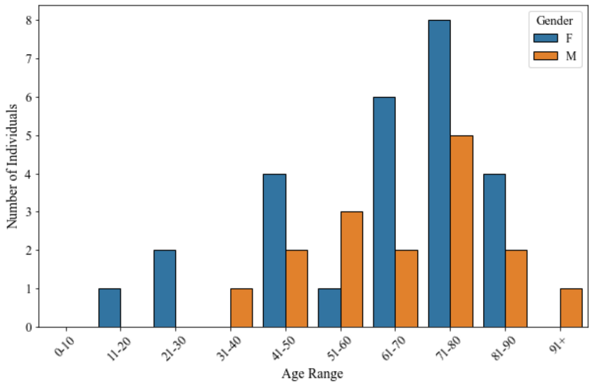

Fig. D7: Illustration of the sex and age distribution of subjects in the BIDMC Dataset. We utilized 42 distinct ECG-PPG pairs from the BIDMC dataset.

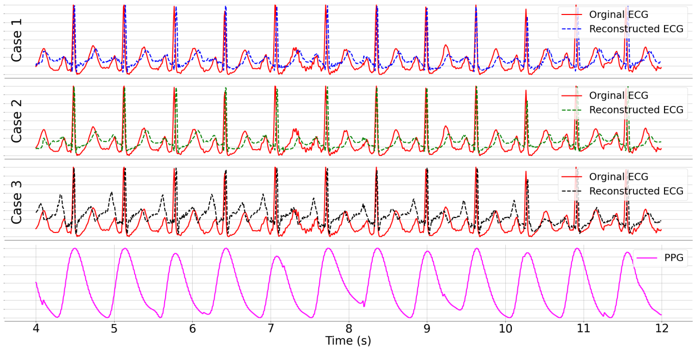

(a) Signal 14.

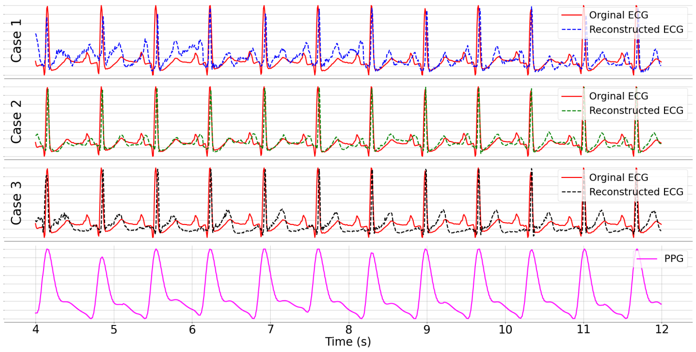

(b) Signal 30.

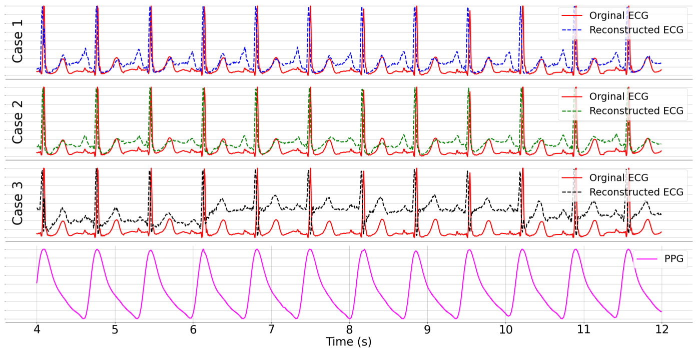

(c) Signal 51.

Fig. D8: ECG reconstruction on female subjects using the proposed CLEP-GAN method: a selection of three random ECG-PPG pairs from female subjects were used as the testing data. Case 1 highlights reconstructed ECGs achieved when exclusively training the model using data from female subjects. Case 2 demonstrates the reconstructed ECGs when the model undergoes a two-step process: initial pretraining on our synthetic dataset followed by fine-tuning exclusively with data from female subjects. Case 3 illustrates the reconstructed ECGs obtained when the model is trained utilizing data from both female and male subjects.
